# Dual-functional Memory and Threshold Resistive Switching Based on the Push-Pull Mechanism of Oxygen Ions

**DOI:** 10.1038/srep23945

**Published:** 2016-04-07

**Authors:** Yi-Jen Huang, Shih-Chun Chao, Der-Hsien Lien, Cheng-Yen Wen, Jr-Hau He, Si-Chen Lee

**Affiliations:** 1 Graduate Institute of Electronics Engineering, National Taiwan University, Taipei 10617, Taiwan; 2 Department of Materials Science and Engineering, National Taiwan University, Taipei 10617, Taiwan; 3 Computer, Electrical and Mathematical Sciences and Engineering (CEMSE) Division, King Abdullah University of Science & Technology (KAUST), Thuwal 23955-6900, Saudi Arabia; 4 Taiwan Consortium of Emergent Crystalline Materials, Ministry of Science and Technology, Taipei 10617, Taiwan

## Abstract

The combination of nonvolatile memory switching and volatile threshold switching functions of transition metal oxides in crossbar memory arrays is of great potential for replacing charge-based flash memory in very-large-scale integration. Here, we show that the resistive switching material structure, (amorphous TiO_x_)/(Ag nanoparticles)/(polycrystalline TiO_x_), fabricated on the textured-FTO substrate with ITO as the top electrode exhibits both the memory switching and threshold switching functions. When the device is used for resistive switching, it is forming-free for resistive memory applications with low operation voltage (<±1 V) and self-compliance to current up to 50 μA. When it is used for threshold switching, the low threshold current is beneficial for improving the device selectivity. The variation of oxygen distribution measured by energy dispersive X-ray spectroscopy and scanning transmission electron microscopy indicates the formation or rupture of conducting filaments in the device at different resistance states. It is therefore suggested that the push and pull actions of oxygen ions in the amorphous TiO_x_ and polycrystalline TiO_x_ films during the voltage sweep account for the memory switching and threshold switching properties in the device.

In the near future, the present charge-based flash memories will face the limits to their applications in the very-large-scale integration (VLSI) technology^1^,^2^,^3^. In order to have compatible storage devices with future high-frequency electronics, several types of non-volatile memory have been developed, such as ferroelectric random access memory (FRAM)^4^,^5^,^6^, magnetoresistive RAM (MRAM)^7^,^8^,^9^ and phase-change RAM (PRAM)^10^,^11^,^12^,^13^, etc. Among these new memory technologies, resistive random access memory (RRAM) has several properties outweighing the transistor-type memories, such as high-speed operation (sub-ns), low power consumption (<0.1 pJ), and high endurance (>10^12^ cycles)^14^,^15^,^16^,^17^,^18^,^19^,^20^,^21^,^22^; it is therefore the most promising candidate to overcome the technological limitations for next-generation non-volatile memory. More importantly, the simpler structure of RRAM makes it feasible to be integrated into a passive crossbar array of high-density memory cells with a cell size of 4F^2^ (F = minimum feature size;). By stacking up the crossbar arrays, a higher density of memory devices can be achieved^23^. Nonetheless, the sneak current through neighboring cells is still a problem in the crossbar array structure. Thus, selector devices are needed in the circuit to suppress the sneak current paths^24^,^25^. In order to replace conventional charge-based transistor selectors, which require a large space on the chip, for future memory technology in VLSI, smaller bi-directional selector devices, such as the mixed-ionic-electronic-conduction device^26^,^27^, bipolar nonlinear device^28^,^29^, and the Schottky diodes^30^,^31^, have recently been incorporated into bipolar RRAM devices.

A material with the resistive switching (RS) characteristics can be used for non-volatile memory switching (MS) or volatile threshold switching (TS). The low-resistance state (LRS) or the high-resistance state (HRS) of the material can be stably maintained without an external sustaining voltage. This property provides attractive prospects for non-volative RRAM applications. When the resistive switching material is used for TS applicaitons, the “ON” and “OFF” states of the device are switchable by adjusting the voltage^32^,^33^. Such TS devices typically exhibit bi-directional switching property, useful for suppressing the sneak current paths between memory cells^34^,^35^,^36^,^37^,^38^,^39^,^40^. Recently, it is found that the MS and TS operations can be realized in a single device structure^32^,^33^,^41^,^42^. When such a dual-functional device is operated in the MS mode, it is used for the non-volatile memory storage; when it is operated in the TS mode, it is a bi-directional selector device. The flexibility and simplicity for the MS and TS applications in a single device is beneficial for the advancement of integrated-circuit design.

In this paper, we show that the memory switching and threshold switching can both be operated in a particular (amorphous TiO_x_)/(Ag nanoparticles)/(polycrystalline TiO_x_) structure sandwiched between indium tin oxide (ITO) and fluorine-doped tin oxide (FTO) electrodes. When this device is used for memory switching, it exhibits good electrical properties, especially, the self-compliant driving current, which is beneficial for ultralow programming energy in RRAM applications. When an external current compliance is applied, the device can be operated for threshold switching. It is then an excellent bi-directional selector for high-density memory applications. In order to understand the electrical conduction mechanism of this device, we use scanning transmission electron microscopy (STEM) and energy dispersive spectroscopy (EDS) to study the compositional distribution in the devices at different resistance states. The results suggest that, under voltage sweeps, the push and pull actions on the oxygen ions in the amorphous TiO_x_ layer and polycrystalline TiO_x_ layer can account for both the memory switching and threshold switching operations in the device.

## Results

### Dual-functional resistive switching device structure and its electrical properties

**Figure 1:**
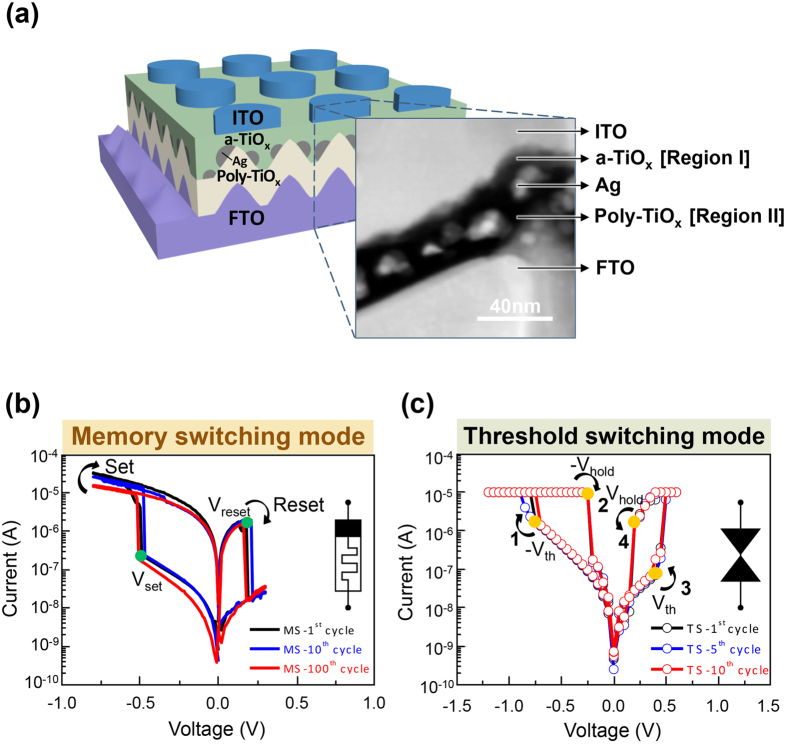
Device structure and electrical properties of the dual functional switching device. (**a**) Schematic illustration of the (amorphous TiO_x_)/(Ag nanoparticles)/(polycrystalline TiO_x_) resistive switching material fabricated on the textured-FTO (fluorine doped tin oxide) glass substrate with ITO (indium tin oxide) as the top electrode. The inset shows the cross-sectional scanning transmission electron microscopy (STEM) image. (**b**) Current-voltage (I-V) curves of the device operated in the memory switching mode of the first, 10^th^, and 100^th^ cycles, respectively. V_set_ and V_reset_ are respectively the setting and resetting voltages used for memory switching. **(c)** I-V curves of the device operated in the threshold switching mode of the first, 5^th^, and 10^th^ cycles, respectively. V_th_ (−V_th_) is the voltage to switch the device to the ON state, and V_hold_ (−V_hold_) is the voltage to switch the device to the OFF state.

The active layer of the dual-functional switching device is composed of an amorphous TiO_x_ layer, Ag nanoparticles, and a polycrystalline TiO_x_ layer. This active layer is fabricated on the textured-FTO bottom electrode, followed by the deposition of the ITO top electrode (Details of device fabrication can be found in Methods). Figure 1(a) shows the schematic illustration and the cross-sectional STEM image of the device structure. The as-deposited device is already in the high-resistance state, so that the bias-assisted forming process is not required for the resistive switching operation^43^.

Figure 1(b) shows the typical current-voltage (I-V) curves of the device in the memory switching mode. When a negative voltage sweep (0 V to −0.8 V) is applied on the top electrode, the current increases rapidly at the SET voltage (V_set_ ≈ −0.5 V); the device is switched from the high resistance state (HRS) to the low resistance state (LRS). When the voltage is swept reversely to 0.3 V, the device is switched back to HRS at the RESET voltage (V_reset_ ≈ 0.2 V). This memory-switching characteristic sustains after 100 switching cycles. It is noted that, when the voltage is more negative than V_set_, the current obeys linear I-V relation (Supplementary Fig. S1(a)) up to 50 μA. Without such a self-compliance property, an external current limit (so-called compliance current) is required. Otherwise, a large current may be produced to cause eternal device failure, so called the hard breakdown, during the forming or setting process. An RRAM device with the self-compliance property is beneficial for the development of high-density memory array or 3D RRAM applications, because an extra transistor-type current-compliance unit is no longer required.

**Figure 2:**
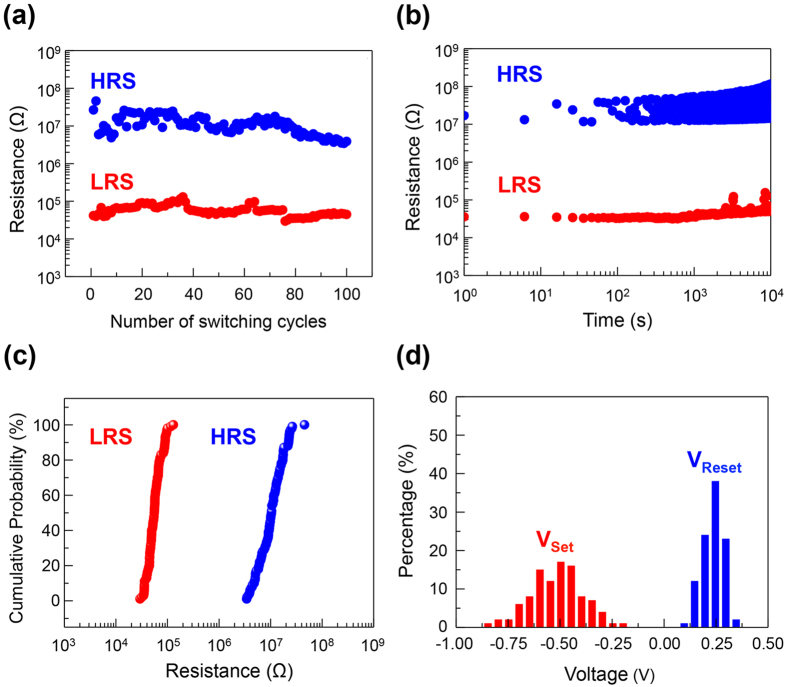
Electrical characteristics of memory switching device of the resistive switching material. (**a**) Resistance of the device at HRS and LRS at different switching cycles. (**b**) Resistances of the devices at HRS and LRS vs. time. The retention of the resistance state is at least 10^4^ s. (**c**) Cumulative probability plots from (**a**) for the resistances of the device at HRS and LRS, respectively. (**d**) Distribution of the set and reset voltages in 100 successive cycles. V_set_ is −0.52 ± 0.13 V, and V_reset_ is 0.24 ± 0.05 V.

When a current compliance (I_cc_ = 10 μA) is subsequently applied on the device of Fig. 1(b), the I-V relation shows the characteristics of threshold switching mode, as shown in Fig. 1(c) (linear I-V relations are plotted in Supplementary Fig. S1(b)). The device is switched from the OFF state to the ON state, when the voltage is swept to higher than −V_th_ = −0.8 V (step 1). As the voltage is swept reversely, the current follows the compliance current until the hold voltage, −V_hold_ = −0.2 V (step 2). The device is then switched back to the OFF state. Similar switching phenomenon occurs in the positive voltage sweep (steps 3 and 4). This threshold-switching characteristic remains similar after 10 successive cycles. In principle, V_th_ in Fig. 1(c) and V_set_ in Fig. 1(b) should be identical. However, there is distribution of the values of V_set_ (as will be shown in Fig. 2(d)). The variation of the value of V_th_ from V_set_ is still within the range of the distribution.

From Fig. 1(c), assuming that a reading voltage is applied between −V_th_ and −V_hold_, the on/off ratio of the selector device is higher than 10. It is also noted that the TS device has low threshold currents, −I_th_ = −2 μA and I_th_ = 0.1 μA, at −V_th_ = −0.8 V and V_th_ = 0.4 V, respectively. Once the TS device is in series with a memory device (so-called 1S + 1R structure) in a memory array, reading memory cells can be accurate for that the problem of sneak current is reduced^39^,^40^. Figure 1(b,c) demonstrate that the device in Fig. 1(a) can be operated in both the memory switching and threshold switching modes; in addition, when it is operated in the threshold switching mode, it is a bi-directional selector.

### Electrical performance for the memory switching and threshold switching operations

The reliability of the resistive switching device in the MS mode is demonstrated in its cycling endurance and retention time tests, as shown respectively in Fig. 2(a,b). In 100 sweeping cycles, the resistances at HRS and LRS, read at −0.1 V, are consistently in a ratio of ~200, as shown in Fig. 2(a). The device is therefore very suitable for memory applications. Figure 2(b) shows the retention of the device at both HRS and LRS at room temperature for 10^4^ s with the reading voltage of −0.1 V. The resistances of the device at HRS and LRS are also uniform, as shown in Fig. 2(c), with mean values of 12 MΩ and 60 KΩ, respectively. The variation of the SET and RESET voltages of this device are also small (Fig. 2(d)); V_set_ is −0.52 ± 0.13 V, and V_reset_ is 0.24 ± 0.05 V.

**Figure 3:**
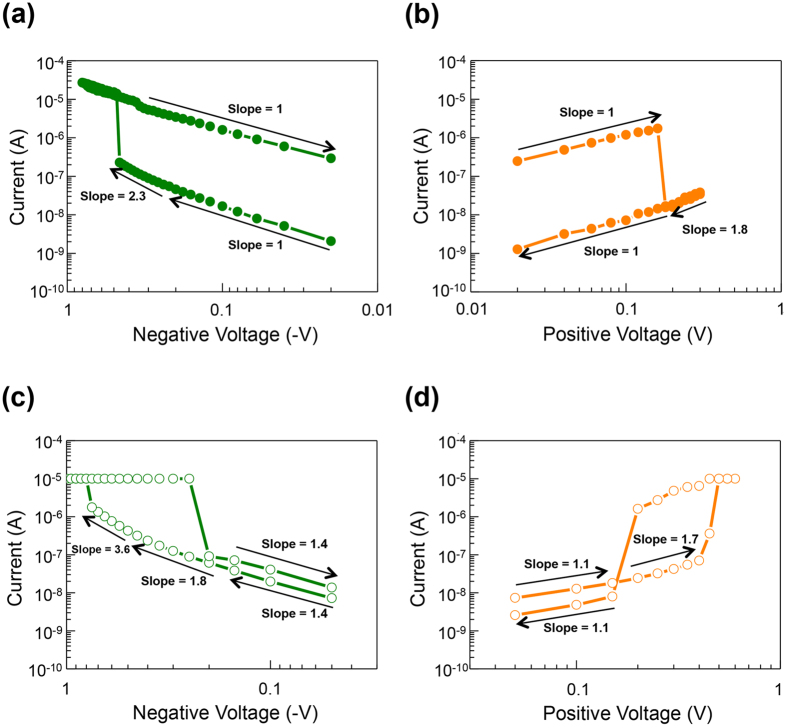
Electrical characteristics of the resistive switching device in the memory switching and threshold switching modes. (**a**,**b**) Double-logarithmic plots of I-V curves of the memory switching operation in the negative and positive bias regions, respectively. (**c**,**d**) Double-logarithmic plots of I-V curves of the threshold switching operation in the negative and positive bias regions, respectively.

To understand the conduction mechanism in the memory switching mode, the I-V curves of the device, (amorphous TiO_x_)/(Ag NPs)/(polycrystalline TiO_x_) with ITO and textured-FTO as the electrodes, in both SET (negative voltage region) and RESET (positive voltage region) processes are plotted respectively in Fig. 3(a,b) in double-logarithmic scale. In the SET process of the device at HRS (Fig. 3(a)), the current varies linearly with voltage (I 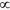 V) in the small bias region, followed by the quadratic dependence (I 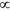 V^2^) in the larger bias region. Such I-V relation is in agreement with the characteristics of the trap-controlled space-charge-limited current^44^,^45^,^46^,^47^, and the dominating trap sites in this device are the oxygen vacancies in the structure. After the SET process, the conducting filament is formed. Thus, the I-V relation presents the ohmic conduction behavior (I 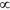 V) at LRS. A similar conducting mechanism is also noted in the RESET process, as shown in Fig. 3(b). A linear I-V relation appears at LRS, and the space-charge-limited current again dominates the conduction after the RESET process.

When operated in the threshold switching mode, the device at HRS in the turn-on process exhibits a nearly linear relation at low voltage regions, in both negative and positive sweeping (Fig. 3(c,d)), followed by a more rapidly increasing current in higher voltage regions. A steeper increase of current is noticed between −0.5 V to −0.8 V (Fig. 3(c)). These features are also in agreement with the trap-controlled space-charge-limited current model^44^,^45^. After the device is turned off, the device exhibits the ohmic conduction property. Because of the similarities in current-voltage characteristics between the memory switching and threshold switching operations, it is believed that the mechanisms of device operation in both modes are similar.

### Composition redistribution during memory switching of the device

**Figure 4:**
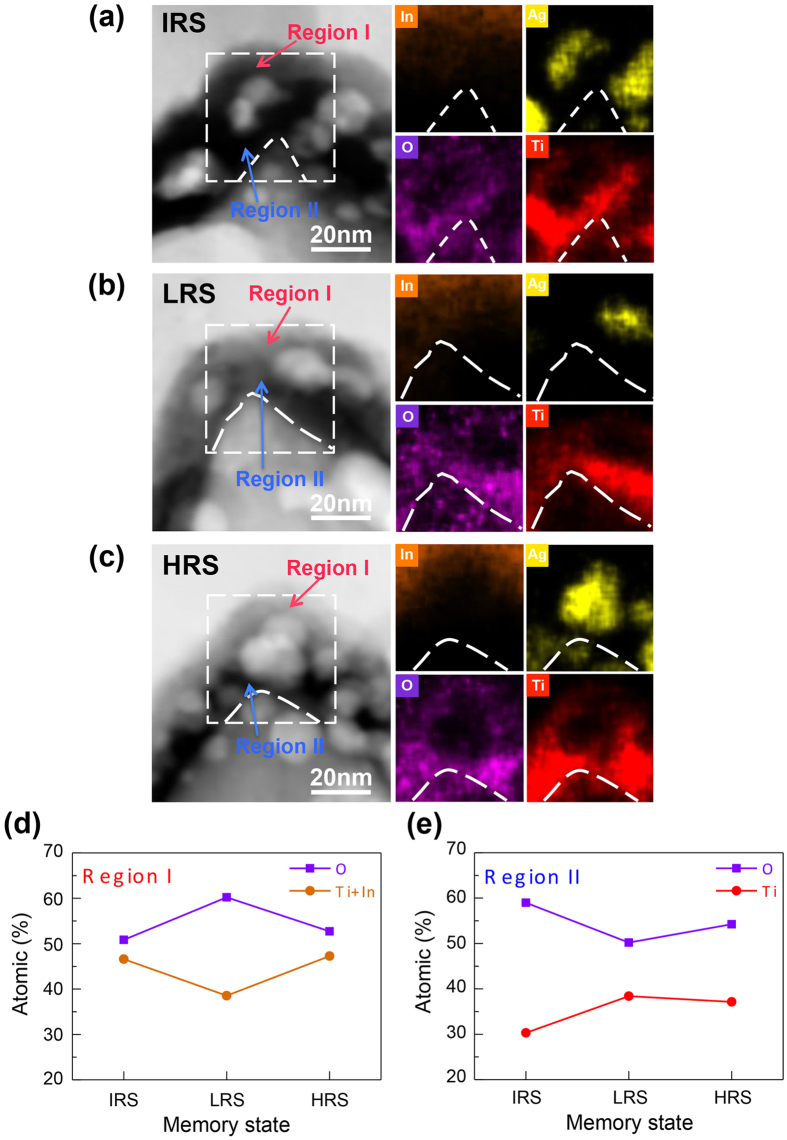
STEM-EDS analysis of the resistive switching cell at different resistance states. The cross-sectional STEM images and the EDS compositional maps of the resistive switching cell at (**a**) the initial resistance state (IRS), **(b)** the low resistance state (LRS), and **(c)** the high resistance state (HRS). The EDS maps of In, Ag, O, and Ti are measured from the indicated area in each STEM image. Region I is the area between Ag nanoparticles and the top ITO electrode in the amorphous TiO_x_ layer. Region II is the area between the bottom FTO electrode and the Ag nanoparticles in the polycrystalline TiO_x_ layer. **(d**,**e)** Plots of the atomic ratio of oxygen in region I and region II, respectively, at different resistance states. These values are calculated from the EDS spectra probed in these regions.

The as-prepared device at the initial resistance state (IRS) and those after the SET process (LRS), and after the RESET process (HRS) are analyzed using STEM. The voltage sweeps, and the corresponding I-V curves, for making the LRS and HRS samples are shown in Supplementary Fig. S2. Figure 4(a–c) displays the STEM high-angle annular dark-field (HAADF) images and the EDS analysis of the three samples. In the HAADF-STEM images, the intensity is sensitively proportional to Z^1.7^, where Z is the atomic number, revealing compositional information; therefore, the top and bottom electrodes and the Ag nanoparticles appear bright, while the TiO_x_ layers are darker. It is also noted that the area of the top amorphous TiO_x_ layer is brighter than that of the bottom polycyrstalline TiO_x_ layer, implying the existence of heavier elements other than Ti and O. From the EDS composition maps of indium in Fig. 4(a–c), the brighter contrast in the amorphous TiO_x_ layer is due to the diffusion of indium from the top ITO electrode. Therefore, the top amorphous TiO_x_ layer should contain a noticeable amount of indium during resistive switching.

It has been reported that, in the TiO_2_-based RRAM devices, the resistive switching is via the formation or rupture of the Magnéli phase (Ti_n_O_2n−1_; n = 3, 4, 5…), which is oxygen deficient and exhibits n-type semiconducting properties^18^,^48^,^49^. When oxygen ions migrate out of the resistive switching layer due to an applied electric field, oxygen vacancies accumulate to form filaments (channels) of the oxygen-deficient TiO_x_ channel. On the other hand, when oxygen ions migrate into the resistive switching layer, the oxygen vacancies are being filled. Therefore, the conducting filaments shrink, making the resistive switching layer more insulating. Indium oxide also exhibits similar resistive switching properties^50^. It is therefore suggested that, in the RRAM devices of Fig. 4, the amorphous TiO_x_ together with indium oxide play the role as the resistive switching layer.

In this study, textured-FTO substrates are used as the bottom electrodes. The titanium oxide resistive switching layer, deposited by ALD (Methods), conformally covers the bottom electrode. The textured-FTO substrate can also enhance the electrical field near the tips on the surface^51^,^52^. When a voltage is applied on this structure, the electric field around the metal particles is also enhanced in the direction parallel to the voltage. Therefore, the electric field between the tips of bottom FTO electrode and the Ag nanoparticle is enhanced to form or rupture the conducting filaments^53^,^54^,^55^. Likewise, the electric field between the Ag nanoparticles and top electrode is also enhanced. As a result of the migration of oxygen ions under the enhanced electric fields, the relative content of oxygen in the regions surrounding the Ag nanoparticles varies at different resistance states as shown in Fig. 4. In such a device structure, Au is also one of the candidates for the nanoparticles. Under similar growth conditions to the Ag nanoparticles in this study, the average particles size of Au nanoparticles is about 16 nm, smaller than that of the Ag nanoparticles, and the density of Au nanoparticles is larger. The thickness of the TiO_x_ resistive switching layer between the Au nanoparticles and the ITO electrode in the device is thicker, so that the effect of field enhancement is weaker. A larger voltage is required to operate the device. Besides, due to the density of Au nanoparticles is higher, the number of conducting filaments is larger, leading to larger currents (Supplementary Fig. S3) and larger power consumption than those shown in Fig. 1(b). For the device without the metal particles, the forming step for memory switching causes the formation of pores in the TiO_x_ layer (Supplementary Fig. S4), and the endurance and reliability are degraded.

Representative composition ratios of O over the transition metals (Ti and In), calculated from the EDS spectra (Supplementary Fig. S5) of the top amorphous TiO_x_ layer (region I) and the bottom poly-crystalline TiO_x_ layer (region II) at each resistance state are plotted in Fig. 4(d,e). At IRS (Fig. 4(a)), the oxygen is deficient (O/(Ti+In) = 1.1) in region I, implying the existence of conducting filament. By contrast, the O/Ti ratio is about 2 in region II, making the layer less conducting. When a negative bias is swept to the setting voltage V_set_, the device is set to LRS (Fig. 4(b)) (Supplementary Fig. S2(a)). The negative bias pushes oxygen ions from the top ITO electrode to region I, and the O/(Ti+In) ratio increases to 1.6. At this process, recombination of oxygen ions with vacancies leads to the rupture of the conducting filaments, so that region I at LRS is less conductive than that at IRS. In region II, the relatively positive bias on the FTO bottom electrode pulls oxygen ions from region II to the electrode, making region II (O/Ti = 1.3) at LRS more conductive than that at IRS.

Reversed movement of oxygen ions occurs when a positive bias is swept over the resetting voltage, V_reset_ (Supplementary Fig. S2(b)), making the device at HRS (Fig. 4c). At this state, some of the oxygen ions in region I have been pulled back to the ITO top electrode, and region I becomes more oxygen-deficient (O/(Ti+In) = 1.1) and more conductive than that at LRS. Meanwhile, oxygen ions are pushed into region II from the FTO bottom electrode, making region II less oxygen-deficient (O/Ti = 1.5) and more insulating than that at LRS.

## Discussion

**Figure 5:**
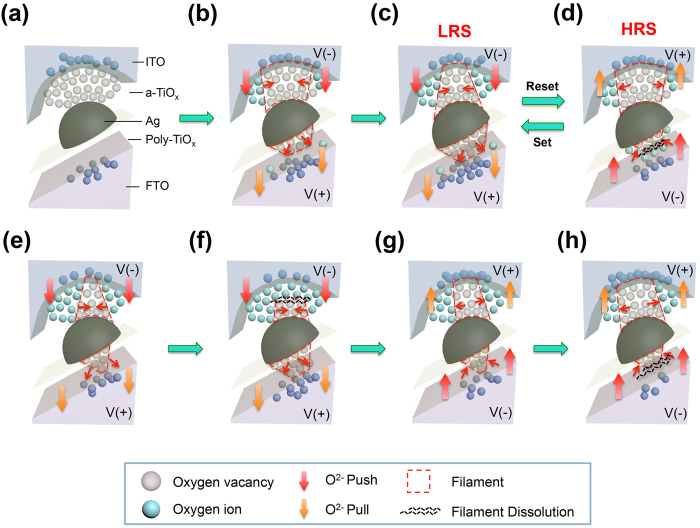
Push-pull mechanism of oxygen ions migration in the memory and threshold switching modes. (**a–d**) Schematic illustrations of the migration of the oxygen ions due to the applied voltage. After device preparation, the concentration of oxygen vacancies (grey circles) at the initial resistance state (IRS) is high in the amorphous TiO_x_ layer (**a**). After applied a negative voltage, oxygen ions (blue circles) are pushed (indicated by red arrows) into the amorphous TiO_x_ (a-TiO_x_) layer and recombine with the oxygen vacancies (**b**). Thus, the filament (the dashed box) in the a-TiO_x_ layer shrinks. In the polycrystalline TiO_x_ (poly-TiO_x_) layer, oxygen ions are pulled (indicated by orange arrows) to the bottom electrode. Filaments are therefore formed in both a-TiO_x_ and poly-TiO_x_ layers, and the cell is switched to the low-resistance state (LRS) (**c**). When a positive voltage is applied, oxygen ions in the a-TiO_x_ layer are pulled out to the top electrode. Meanwhile, some oxygen ions are pushed from the bottom electrode to the poly-TiO_x_ layer. Recombination of oxygen ions with the vacancies in the poly-TiO_x_ layer causes dissolution of the filaments, indicated by the black break lines in (**d**). The sample is at the high-resistance state (HRS). **(e–h)** Schematic illustrations of the migration of oxygen ions to switch the device to the ON state (**e**,**g**) and the OFF state (**f**,**h**).

According to the STEM-EDS analysis results in Fig. 4, the resistive switching of device is due to the push-pull mechanism of the oxygen ions in the top amorphous TiO_x_ layer and the bottom polycrystalline TiO_x_ layer under enhanced electrical fields. Figure 5(a–d) schematically explains the migration of oxygen ions during the setting and resetting steps. In the as-prepared device, Fig. 5(a), the top amorphous TiO_x_ region is oxygen-deficient, with many conducting filaments, and the bottom polycrystalline TiO_x_ is oxygen-rich, without conducting filaments (according to the EDS results of the IRS sample in Fig. 4(a)).

During the SET process (Fig. 5(b)), a negative voltage sweep pulls the oxygen ions from the polycrystalline TiO_x_ layer to the bottom electrode. Once the oxygen vacancies form a conducting filament, the current increases abruptly and the device is switched to LRS. Meanwhile, a large amount of electrons are injected from top ITO electrode into the amorphous TiO_x_ film. These electrons enhance (push) the oxygen ions migration from the top ITO electrode to the amorphous TiO_x_ layer. The oxygen ions recombine with oxygen vacancies, so that the existing conducting filament in this layer shrinks. As a result, the resistance in the top amorphous TiO_x_ layer increases rapidly, leading to the self-compliance current (Fig. 5(c)). Thus, the device presents ohmic I-V relation, as shown in Fig. 3(a), and hard breakdown of the device is avoided.

During the RESET process, Fig. 5(d), the positive voltage sweep pulls the oxygen ions from the amorphous TiO_x_ layer to the top ITO electrode, and pushes the oxygen ions from the bottom FTO electrode into the polycrystalline TiO_x_ layer. Even though the conducting filament grows in the amorphous TiO_x_ layer (as evidenced in Fig. 4(c), the oxygen concentration in region I decreases), the filaments in the polycrystalline TiO_x_ layer shrink, making the device at HRS. The asymmetric I-V relation present in this device is perhaps due to the difference between the oxygen migration rates across the ITO/amorphous TiO_x_ and polycrystalline TiO_x_/FTO interfaces.

To operate the device for threshold switching, the current compliance is required; furthermore, the range of voltage sweep is wider in threshold switching than that in memory switching. For setting the device to the ON state (step 1 in Fig. 1(c)), the negative voltage sweep drives the migration of oxygen ions to form filaments in the polycrystalline TiO_x_ (Fig. 5(e)). When the bias is reduced, step 2 in Fig. 1(c), the current is still maintained at the compliance current. The current flow through the top ITO electrode also causes the migration of oxygen ions to fill the oxygen vacancies in the amorphous TiO_x_ layer. In contrast to memory switching, the prolonged migration of oxygen ions and accompanied Joule heating rupture the existing conducting filaments, and the device is switched to the high resistance OFF state (Fig. 5(f)). The device is therefore has the volatile memory property. The positive voltage sweep also switches the device to the ON and OFF states (steps 3 and 4 in Fig. 1(c)) in similar mechanisms, as shown in Fig. 5(g,h), respectively.

In summary, dual-functional resistive switching devices for nonvolatile memory switching and volatile threshold switching applications can be realized in a structure composed of (amorphous TiO_x_)/(Ag NPs)/(polycrystalline TiO_x_) with ITO and textured-FTO as the electrodes. The resistive switching is due to the formation and rupture of conducting filaments in the TiO_x_ layers between Ag nanoparticles and the electrodes, where the electric fields are enhanced. Migration of oxygen ions due to the enhanced electric fields is observed in the STEM-EDS composition analysis. Based on the push-pull mechanism, the migration of oxygen ions accounts for the formation and rupture of the conducting filaments, which are made of oxygen-deficient TiO_x_ structure. This resistive switching device is forming-free, with low (<±1 V) and uniform setting and resetting voltages. It also has uniform resistance at HRS and LRS and the self-compliant current-voltage relation for RRAM applications. By applying a compliance current (10 μA), the device is operated in the threshold switching mode and can be a bipolar selector with a high on/off ratio.

## Methods

### Device fabrication

A 10 nm amorphous titanium oxide (TiO_x_) thin film was deposited on a textured-FTO (~300 nm) on glass substrate using atomic layer deposition (ALD) with titanium isopropoxide (Ti(OCH(CH_3_)_2_)_4_, TTIP) as the titanium precursor and H_2_O as the oxygen precursor at 200 °C. Then the sample was annealed at 500 °C in oxygen for 5 min using the rapid thermal annealing (RTA) process. During the RTA process, the amorphous TiO_x_ thin film transforms into the polycrystalline anatase TiO_x_ phase.

A 3 nm thick Ag film was thermally evaporated onto the polycrystalline TiO_x_ thin films at a rate of 0.1 Å/s in the vacuum of 4 × 10^−6^ Torr. The subsequent annealing at 300 °C for 5 min in nitrogen aggregated the Ag films to form nanoparticles. The average diameter was approximately 25 nm (Supplementary Fig. S6). Under the same sample growth conditions, the average diameter of Au nanoparticles was around 16 nm (Supplementary Fig. S7).

After the formation of Ag NPs, a 10 nm amorphous TiO_x_ film was deposited onto the sample surface using ALD at 200 °C. The top ITO electrode in a thickness of 100 nm was deposited in a DC Ar plasma sputtering system at 150 °C. The electrodes are in a diameter of 250 μm, defined by a shadow mask during deposition.

### Electrical measurements

The current-voltage curves, cycling endurance, and data retention of the devices were measured by a Keithley 4200 semiconductor parameter analyzer. While a DC voltage was applied to the top ITO electrode, the bottom FTO electrode was grounded. For testing the device in the memory switching mode, no current compliance was needed to avoid the hard breakdown problems, but, in the threshold switching mode, a current compliance was required.

### Sample preparation for scanning transmission electron microscopy analysis

Specimens of the device cells at the initial resistance state (IRS), after switched to the low resistance state (LRS), and after switched back to the high resistance state (HRS) were respectively prepared in a focused ion beam (FIB) system, FEI Helios 600i. STEM imaging and EDS analysis of these specimens were conducted in a 200 kV FEI Tecnai Osiris transmission electron microscope.

## Additional Information

**How to cite this article**: Huang, Y.-J. *et al*. Dual-functional Memory and Threshold Resistive Switching Based on the Push-Pull Mechanism of Oxygen Ions. *Sci. Rep*. **6**, 23945; doi: 10.1038/srep23945 (2016).

## Supplementary Material

Supplementary Information
