# Supplementary material for: Dual-functional Memory and Threshold Resistive Switching Based on the Push-Pull Mechanism of Oxygen Ions
Source: Sci Rep. 2016 Apr 7;6:23945. doi: 10.1038/srep23945 (PMC4823777; doi:10.1038/srep23945)
Supplement: Supplementary Information [file srep23945-s1.pdf]

Supplementary Material for Dual-functional Memory and Threshold Resistive Switching Based on the Push-Pull Mechanism of Oxygen Ions

*Yi-Jen Huang[1], Shih-Chun Chao[2], Der-Hsien Lien[1], Cheng-Yen Wen[2,4], Jr-Hau He[3], and Si-Chen Lee[1,]**

[1]*Graduate Institute of Electronics Engineering, National Taiwan University, Taipei 10617, Taiwan.*

[2]*Department of Materials Science and Engineering, National Taiwan University, Taipei 10617, Taiwan.*

[3]*Computer, Electrical and Mathematical Sciences and Engineering (CEMSE) Division, King Abdullah University of Science & Technology (KAUST), Thuwal 23955-6900, Saudi Arabia.*

[4]*Taiwan Consortium of Emergent Crystalline Materials, Ministry of Science and Technology, Taipei 10617, Taiwan.*

**E-mail : sclee@ntu.edu.tw*

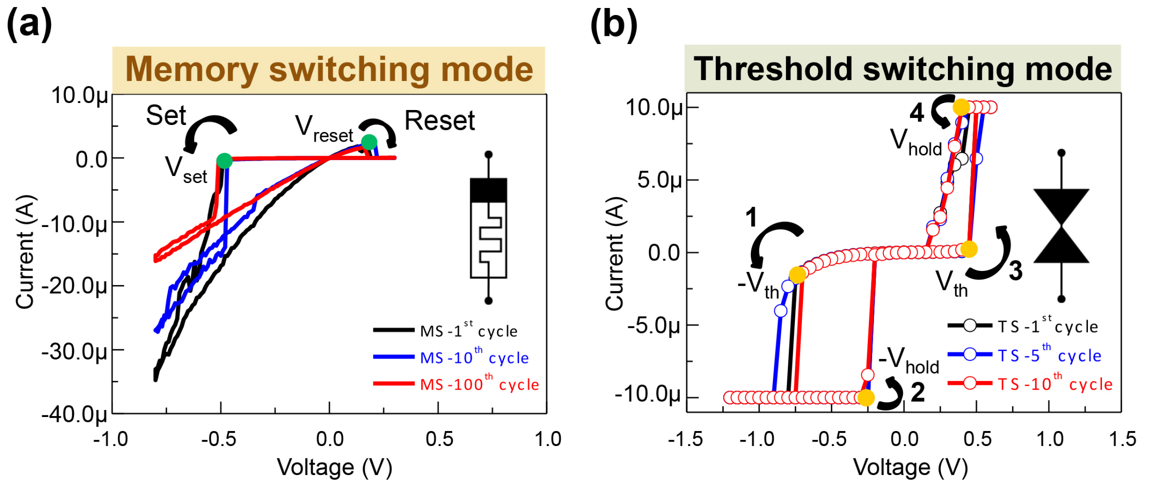

Supplementary Figure S1 | Electrical properties of the dual functional switching device. Linear plot of the current-voltage (I-V) curves of the device in the **(a)** memory switching and **(b)** threshold switching modes.

The as-prepared device at the initial resistance state (IRS) and those after the SET process (LRS), and after the RESET process (HRS) are analyzed using scanning transmission electron microscopy (STEM), Fig. 4. The voltage sweeps, and the corresponding I-V curves, for making the LRS and HRS samples are shown in Fig. S2.

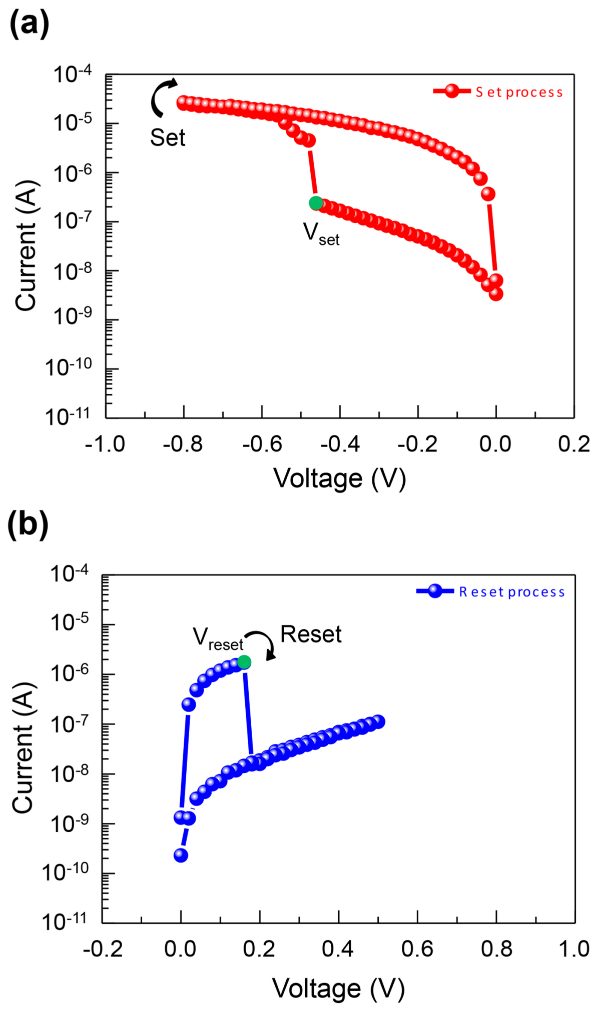

Supplementary Figure S2 | Current-voltage curves during the voltage sweeps to prepare the samples for STEM-EDS analysis. (a, b) D.C. I-V curves recorded during the voltage sweeps to switch the device to the low resistance state and high resistance state for the STEM-EDS in Fig. 4 and Fig. S2.

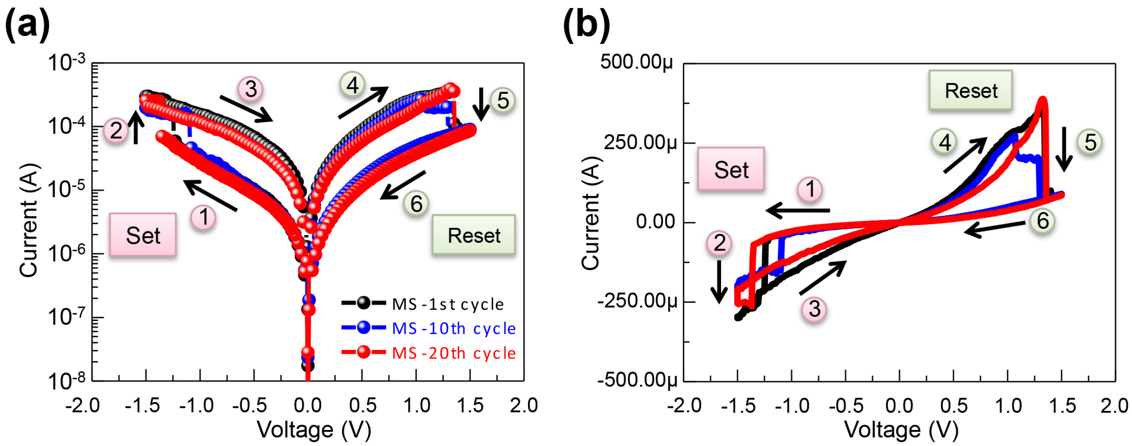

Supplementary Figure S3 | **(a)** Current-voltage (I-V) curves in the memory-switching mode of the device structure (amorphous TiO_x) / (Au nanoparticles) / (polycrystalline TiO_x) resistive switching material fabricated on the textured-FTO (fluorine doped tin oxide) glass substrate with ITO (indium tin oxide) as the top electrode. **(b)** Linear plot of the I-V curves in (a).

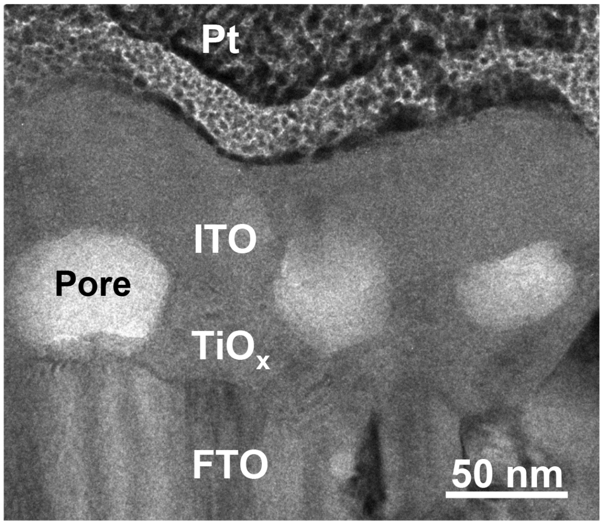

Supplementary Figure S4 | Transmission electron microscopy image of the resistive switching layer in the FTO/TiO_x/ITO device structure after the forming step for the memory switching operation.

The atomic ratio of oxygen in region 1 and region 2 at the IRS, LRS, and HRS are plotted in Fig. 4(d) and (e). The ratios are calculated from the representative EDS spectra.

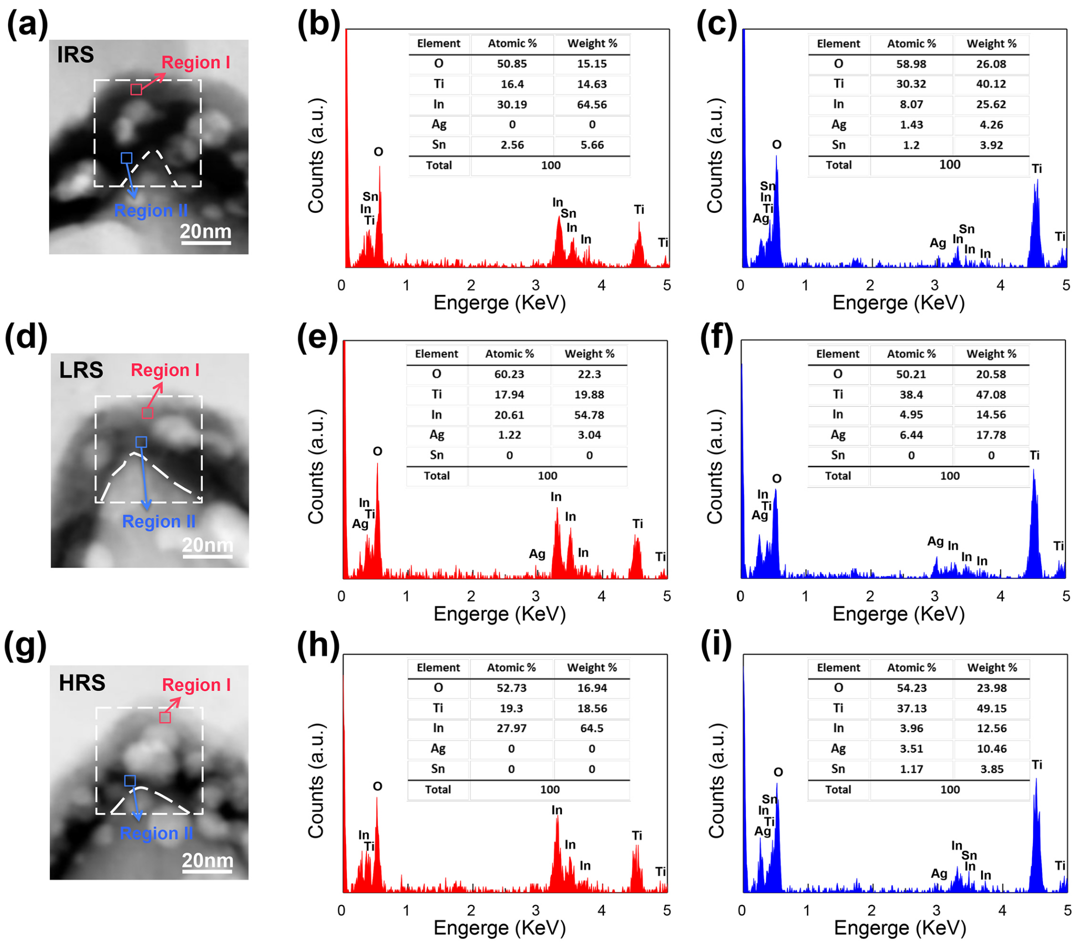

Supplementary Figure S5 | STEM-EDS analysis of the TiO_x layers at different resistance states. (a, d, g) STEM images of the resistive switching device at the initial resistive state (IRS), low resistance state (LRS), and high resistive state (HRS). The scale bars in (**a**, **d** and **g**) are 20 nm. **(b, e, h)** Representative EDS spectra taken from the labeled area in region I at IRS, LRS and HRS, respectively. **(c, f, i)** Representative EDS spectra taken from the labeled area in region II at the three different states, respectively.

The size distribution of silver nanoparticles, which bridge the conducting filaments in the top amorphous TiO_x layer and those in the bottom polycrystalline TiO_x layer are analyzed using scanning electron microscopy, as shown in Fig. S3.

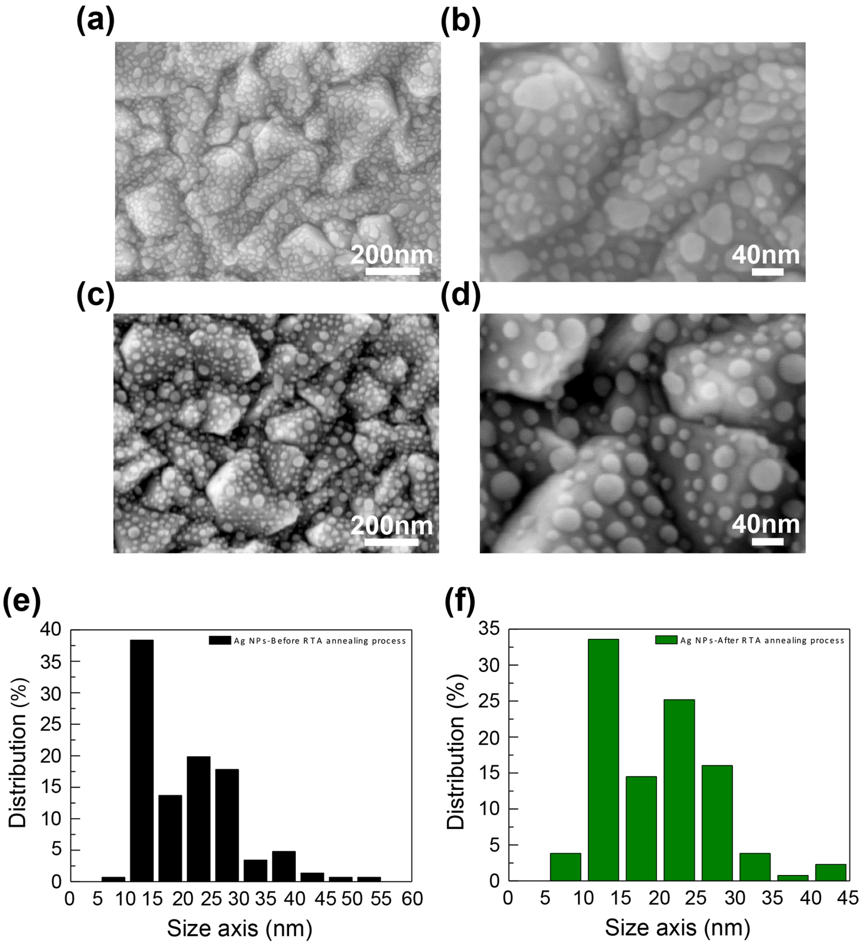

Supplementary Figure S6 | SEM analysis and size distribution of Ag nanoparticles. **(a, b)** SEM images of Ag nanoparticles (NPs) deposited on the textured-FTO electrode. The scale bars are 200 nm in (a) and 40 nm in (b). **(c, d)** SEM images of coalesced Ag NPs after the rapid thermal annealing (RTA) process. **(e, f)** Diagrams of the size distribution of the Ag NPs before and after the RTA process, respectively.

The size distribution of gold nanoparticles, which bridge the conducting filaments in the top amorphous TiO_x layer and those in the bottom polycrystalline TiO_x layer are analyzed using scanning electron microscopy, as shown in Fig. S7.

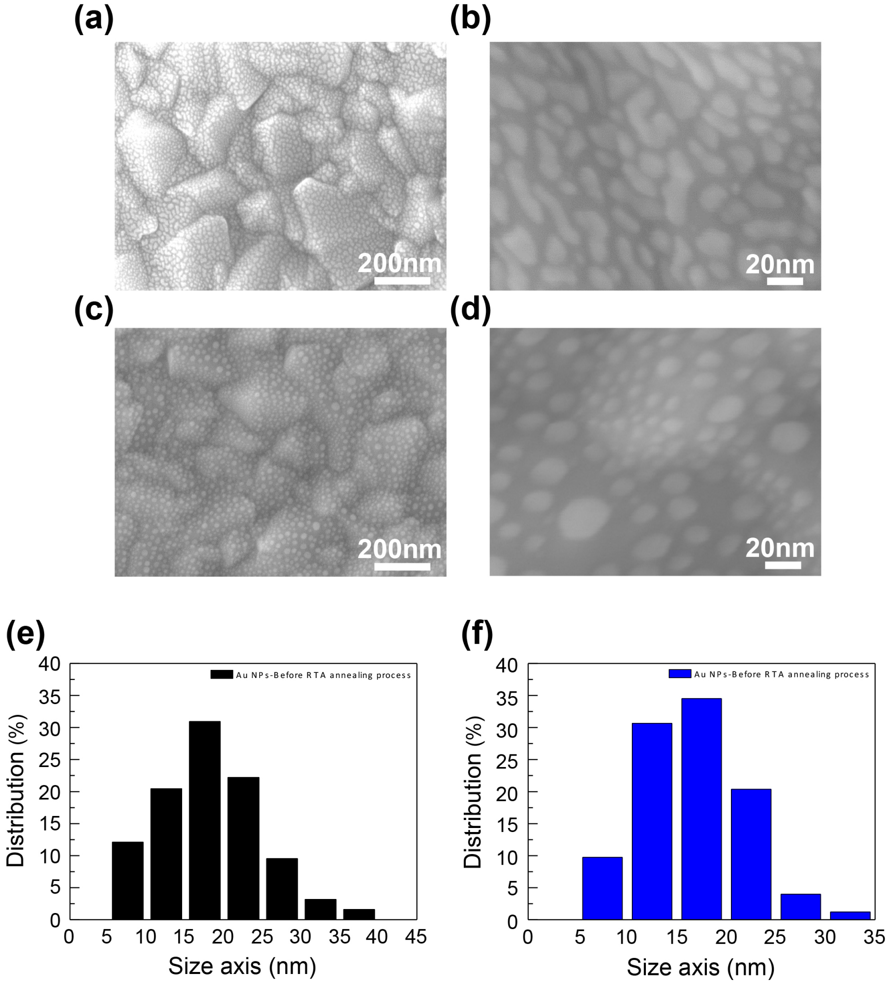

Supplementary Figure S7 | SEM analysis and size distribution of Au nanoparticles. (a, b) SEM images of Au NPs deposited on the textured-FTO electrode. The scale bars are 200 nm in (a) and 20 nm in (b). **(c, d)** SEM images of coalesced Au NPs after the rapid thermal annealing (RTA) process. **(e, f)** Diagrams of the size distribution of the Au NPs before and after the RTA process, respectively.
